# Advances in bacteria‐based drug delivery systems for anti‐tumor therapy

**DOI:** 10.1002/cti2.1518

**Published:** 2024-06-26

**Authors:** Han Shuwen, Song Yifei, Wu Xinyue, Qu Zhanbo, Yu Xiang, Yang Xi

**Affiliations:** ^1^ Huzhou Central Hospital Affiliated Central Hospital Huzhou Universityy Huzhou Zhejiang Province China; ^2^ Huzhou Central Hospital Fifth Affiliated Clinical Medical College of Zhejiang Chinese Medical University Huzhou Zhejiang Province China; ^3^ Key Laboratory of Multiomics Research and Clinical Transformation of Digestive Cancer of Huzhou Huzhou Zhejiang Province China

**Keywords:** anti‐tumor, bacteria, drug delivery system, synthetic biology

## Abstract

In recent years, bacteria have gained considerable attention as a promising drug carrier that is critical in improving the effectiveness and reducing the side effects of anti‐tumor drugs. Drug carriers can be utilised in various forms, including magnetotactic bacteria, bacterial biohybrids, minicells, bacterial ghosts and bacterial spores. Additionally, functionalised and engineered bacteria obtained through gene engineering and surface modification could provide enhanced capabilities for drug delivery. This review summarises the current studies on bacteria‐based drug delivery systems for anti‐tumor therapy and discusses the prospects and challenges of bacteria as drug carriers. Furthermore, our findings aim to provide new directions and guidance for the research on bacteria‐based drug systems.

## Graphical abstract

In this study, we discuss the application of bacteria or bacterial secretions, including magnetotactic bacteria, bacterial biohybrids, minicells, bacterial ghosts, and bacterial outer membrane vesicles, as drug carriers in the delivery of anti‐tumor drugs. Synthetic biology, chemistry and nanotechnology are widely used in bacteria‐based drug delivery systems to enhance tumor‐targeting ability and therapeutic efficacy. However, achieving clinical translation of bacteria‐based drug delivery systems for anti‐tumor therapy is challenging because of biosafety concerns, regulatory hurdles and ethical concerns.

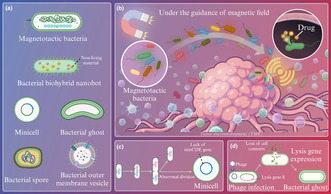

## Introduction

As of 2020, there were approximately 19 292 789 newly reported cancer cases worldwide, with 9 958 133 deaths.^1^ Certain types of cancer exhibit low 5‐year survival rates, including pancreatic cancer (11%), oesophageal cancer (20%) and ovarian cancer (49%).^2^ Surgery, radiotherapy and chemotherapy are frequently employed in clinical treatment, but they possess certain limitations.^3^, ^4^, ^5^ While surgical treatment can provide curative results for early‐stage tumors, more than 70% of patients receive their diagnosis during the intermediate and advanced stages, so they miss the optimal window for surgery.^6^ Consequently, there is an imperative need to explore novel anti‐tumor strategies.

As an emerging method for tumor treatment, nano‐drug delivery system can precisely target tumors and reduce the drug's toxic side effects. Nano‐drugs usually accumulate at the tumor site for the enhanced permeability and retention (EPR) effect.^7^ Common nano‐drug delivery systems (PLGA nanoparticles, liposomes and iron nanoparticles) have been utilised for targeted cancer therapy.^8^, ^9^ However, as a result of the capture of drug carriers by the mononuclear phagocyte system in the liver and spleen, the delivery efficiency of nano‐drugs to solid tumors is only approximately 0.7%.^10^ Moreover, complex intratumor environment can impede drug delivery, thereby diminishing the efficacy of anti‐tumor drugs.^11^, ^12^, ^13^ Therefore, it is imperative to develop a drug delivery carrier that possesses clinical accessibility and ensures high delivery efficiency.

Bacteria are acknowledged as highly promising biological nano‐drug carriers and have gained significant attention. First, bacteria and bacterial derivatives possess the inherent ability to target tumors. Several bacterial species (*Salmonella* Typhimurium, *Listeria* and *Streptococcus*) have demonstrated a propensity for selectively targeting solid tumors in preclinical trials.^14^, ^15^, ^16^, ^17^ Because of the hypoxic, immunosuppressive and eutrophic characteristics of tumor microenvironment, obligate and facultative anaerobes (*Escherichia* and *Clostridium*) can enrich and proliferate at the tumor site.^18^, ^19^, ^20^ Compared with chemically synthesised drug delivery systems, bacteria are expected to overcome the high osmotic pressure of tumors that hinder drug penetration and reduce damage to healthy tissues. Moreover, synthetic biology could facilitate the application of engineered bacteria in drug delivery systems. Under the guidance of engineering ideas, synthetic biology uses multi‐disciplinary techniques, including biology, bioinformatics and computer science to construct controllable genetic, metabolic, or signalling networks to enable cells to synthesise substances that are required for humans.^21^ Bacteria can be genetically modified using CRISPR/Cas9 technology to construct targeted drug delivery platforms.^22^ Exploiting gene circuits further enables bacteria to deliver nano‐drugs precisely, continuously and synchronously to tumor cells.^23^ Furthermore, the innate virulence of bacteria accelerates tumor regression.^24^

Bacteria exhibit potential for delivering anti‐tumor drugs, aiding drug accumulation at the tumor site, preventing drug leakage, and ultimately enhancing therapeutic efficacy against tumors. Bacteria have emerged as a promising carrier for the targeted delivery of anti‐tumor drugs, allowing for enhanced accumulation at tumor sites while minimising drug leakage. In this review, the authors aim to promote the utilisation and advancement of bacteria‐based drug delivery systems in anti‐tumor therapy.

## Bacteria‐based nano‐drug carriers for anti‐tumor therapy

**Figure 1:**
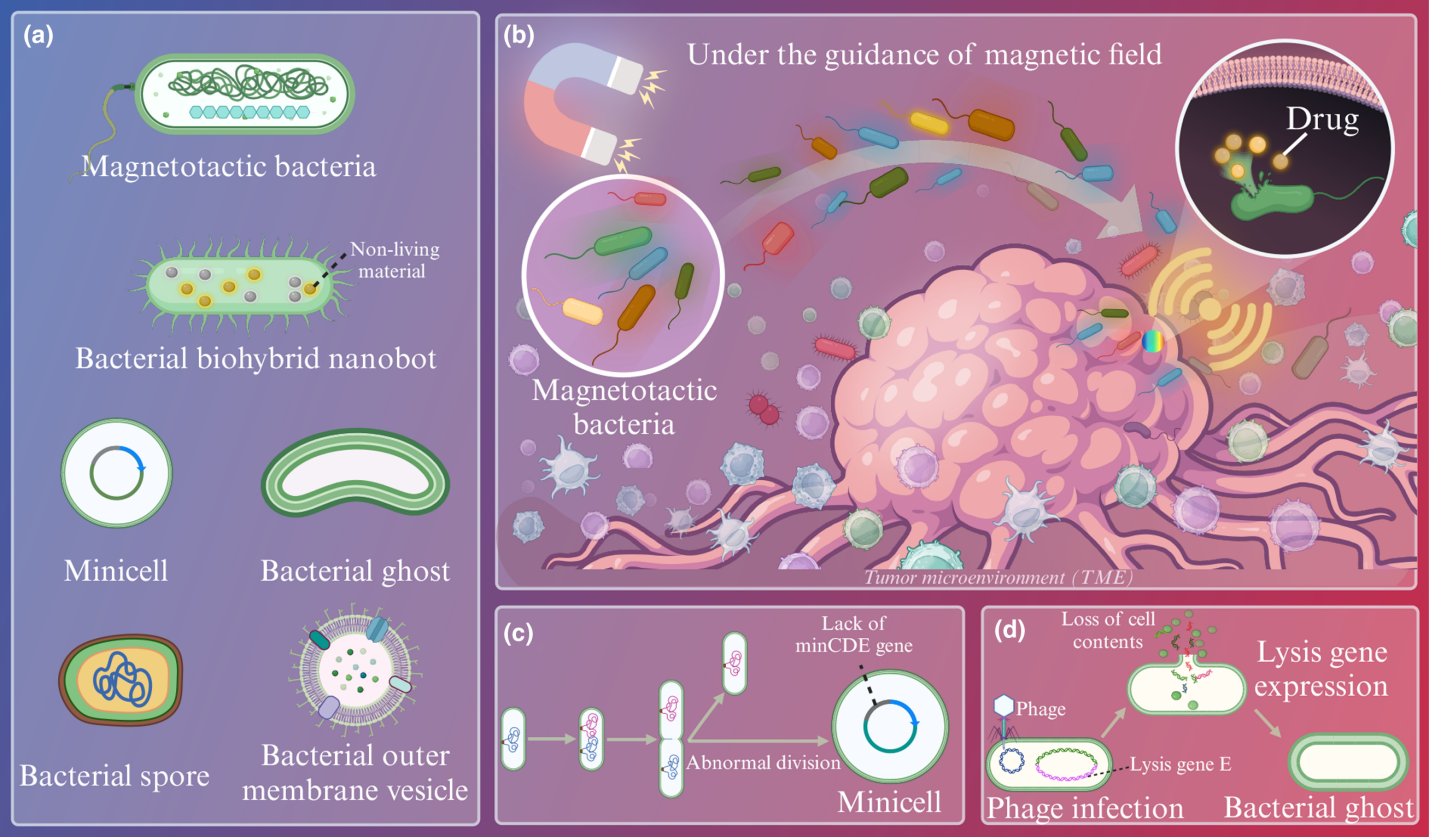
Bacteria‐based nanodrug carrier for anti‐tumor therapy. **(a)** Bacteria act as nanomedicine carriers in various forms, including magnetotactic bacteria, bacterial biohybrid nanobots, minicells, bacterial ghosts, bacterial spores and bacterial outer membrane vesicles, for drug delivery. **(b)** The magnetotactic bacteria can be manipulated to target the tumor site by magnetic field to achieve targeted drug delivery. **(c)** Formation of minicell. **(d)** Formation of bacterial ghost.

Bacteria and bacterial secretions have been widely used as promising drug carriers in anti‐tumor therapy. As carriers, various forms of bacteria, including magnetotactic bacteria, bacterial biohybrid nanobots, minicells, bacterial ghosts, bacterial spores and bacterial outer membrane vesicles (OMV), are expected to improve the efficiency of drug delivery (Figure 1a).

### Magnetotactic bacteria

In recent years, magnetic nanodrugs that are guided by external magnetic field have been considered having the potential to overcome the dense extracellular matrix and high tumor interstitial pressure to improve the efficiency of targeted drug delivery.^25^, ^26^ However, the larger the nanoparticles are, the stronger the magnetic properties are, thus forming aggregates among the magnetic particles and preventing the nanomaterials from penetrating the tumor tissue. Using magnetotactic bacteria as drug carriers can solve the problem that magnetic nano‐drugs penetrating tumor tissue is blocked, and it is expected to improve the efficiency of drug delivery.

Low‐hazard and non‐invasive magnetic fields (MFs) enable the navigation of drug‐carrying bacteria to achieve controlled manipulation of drug delivery^27^, ^28^ (Figure 1b). Bacteria with magnetic properties can independently migrate deep inside the tumor after entering it through the vessel wall. In addition to naturally occurring bacteria, magnetotactic bacteria can also be obtained by biochemical coupling and biomimetic mineralisation for application in MF‐mediated drug delivery systems.^29^, ^30^ Once the tumor is localised by MF, imaging techniques are no longer needed to track the bacteria.^31^ Because of the bacterial magnetosome that contains iron oxide particles, magnetotactic bacteria can be manipulated by external MF,^32^ and they also show high clinical safety,^27^ so magnetotactic bacteria are widely used to enable steerable delivery of drugs.

For instance, without chemical modification, the doxorubicin (DOX)‐internalised MSR‐1 swimming bacteria (MSRD) was constructed by co‐incubation.^33^ When the tumor mice were administered, MSRD achieved the best anti‐tumor effect, which showed the smallest volume and weight of the tumor as well as the highest number of apoptotic tumor cells. MSRD enhances the anti‐tumor effect of DOX, thus holding great promise for spatio‐manipulable drug delivery in precision medicine.

### Bacterial biohybrid nanobots

Bacterial biohybrids, consisting of bacteria and non‐living materials, emerged as a promising approach for drug delivery in anti‐tumor therapy.^34^ The structural composition of the bacterial surface enables the adhesion of non‐living materials through physical and chemical interactions. The teichoic acids found on the cell wall of gram‐positive bacteria create a negatively charged bacterial surface, which is essential for attachment through electrostatic interactions.^35^ Gram‐negative bacteria possess surfaces abundant in hydrophobic lipids, which facilitates the reduction of surface energy by attaching to hydrophobic materials. For example, *Salmonella enterica* serovar Typhimurium (*S*. Typhimurium) rapidly attaches to hydrophobic microcubic surfaces within 5 s.^36^ Next, the high‐affinity interaction between biotin and streptomycin is a common chemical strategy that may require the chemical biotinylation of bacteria via gene engineering.^37^, ^38^

Bacteria can specifically target tumors, allowing bacterial biohybrid nanobots to accumulate at tumor sites. Moreover, bacteria possess the capability to detect environmental stimuli such as pH and temperature, which is vital for the controlled release of drugs.^39^ Consequently, bacteria‐based biohybrid nanorobots exhibit enhanced delivery efficiency and lower therapeutic costs in drug delivery systems compared with non‐living carriers, such as liposomes and nanoparticles.^40^ For example, researchers developed an *Escherichia coli* (*E. coli*)‐based biohybrid nanobot that was loaded with anti‐tumor drugs and magnetic nanoparticles for applications in photothermal therapy.^30^ In 3D CRC tumor spheroids, the combined treatment of near‐infrared (NIR) light irradiation and bacterial biohybrid nanobot treatment results in a remarkable seven‐fold increase in doxorubicin (DOX) uptake by the tumor, with outstanding performance.

### Minicells

Minicells, about 400 nm, are enucleated cells formed by abnormal cell division^41^ (Figure 1c). The absence of the minCDE gene in minicells prevents them from dividing cells and interfering with the normal growth and division of other cells.^42^ Because of their high drug loading capacity, safety and low toxicity, minicells have emerged as a focal point in drug delivery systems.^43^ The drugs released from minicells can effectively reach the hypoxic zone of the tumor, enhancing efficacy and overcoming drug resistance.^44^

In a phase I clinical trial, paclitaxel was delivered using *S.* Typhimurium‐derived minicells, which showed moderate clinical efficacy and safety.^45^ Among the 22 patients who completed at least one cycle of treatment, the minicell‐based delivery system resulted in stable disease in 45%, with only mild self‐limited increases in the inflammatory cytokines IL‐6, IL‐8, TNF‐α and anti‐inflammatory IL‐10 observed.^45^ The minicells generated by *E. coli* Nissle 1917 (*EcN*) were developed to deliver DOX to breast tumor cells, thus resulting in a smaller tumor volume (494 mm^3^) compared with the control group in BALB/c mice with orthotopic 4T1 breast cancers.^44^ Overall, minicell‐based drug delivery systems have a relatively high safety profile and enhanced anti‐tumor effects.

### Bacterial ghosts

Gram‐negative bacteria produce bacterial ghosts (BGs) that are essentially porous empty bacterial shells with about 250 femtoliter per BG intracellular spaces for drug delivery.^46^ The expression of lysis gene E on bacteriophages ΦX174 that is achieved via gene engineering can transform gram‐negative bacteria into BGs^47^, ^48^ (Figure 1d). The protein encoded by gene E leads to the formation of transmembrane tunnel structures that allow the excretion of cytoplasmic contents under osmotic pressure.^49^

Bacterial ghosts are favored as a drug carrier for its advantages compared with the non‐living carriers. The safety of BG has been discovered through *in vivo* and *in vitro* uptake studies.^50^ Afterwards, as a result of the mild production process of BGs, all structural antigens expressed by the bacteria are not destroyed, which allows BGs to retain immunogenicity to stimulate the host's immune system.^51^, ^52^ Compared with liposomes, as carriers loaded with tumor lysates, BGs have achieved better immunotherapy efficacy.^53^ Furthermore, as a natural adjuvant, BGs enhance the immunogenic cell death of tumor cells induced by oxaliplatin.^54^ Finally, BGs are highly stable and can be maintained at ambient temperatures for several months.^55^

### Bacterial spores

In the tumor microenvironment, bacterial spores, a form of survival for bacteria in extremely harsh environments, can adapt to hypoxic conditions.^56^ In the gut, a significant number of bacteria can produce spores, accounting for about 50% of the gut bacteria, among which *Bacillus subtilis* and *Clostridium difficile* have been extensively studied.^56^, ^57^, ^58^, ^59^ Spore growth is highly specific to the tumor site, thus providing bacterial spores a powerful potential as drug carriers.^60^ The strategies to exert the carrier potential of bacterial spores include recombinant and non‐recombinant methods. Recombinant methods rely on coating proteins on the surface of bacterial spores, such as CotB and CotC, which are widely used as anchor proteins in vaccine antigen delivery systems.^61^ The non‐recombinant methods is to attach the desired protein drug to the spore surface by hydrophobic or electrostatic interactions, or by the use of cross‐linking agents, such as glutaraldehyde.^62^

Because of the high stability and resistance of bacterial spores, probiotic spores have great application potential in oral drug delivery systems.^63^, ^64^ The use of spores as carriers can improve the oral efficacy of drugs with poor chemical stability and pharmacokinetics, because drug‐loaded spores can survive in the gut across the stomach.^64^, ^65^ A spore‐based oral drug delivery system (SPORE‐MGEM) was constructed, in which *Clostridium butyricum* is covalently coupled to gemcitabine‐loaded mesoporous silicon nanoparticles (MGEM).^66^ The treatment with SPORE‐MGEM results in an approximately three‐fold increase in intratumoral drug accumulation. Moreover, SPORE‐MGEM treatment inhibits 80% of tumor growth in the orthotopic transplanted pancreatic ductal adenocarcinoma mice model. As a carrier, bacterial spores greatly improve the efficiency of oral drug delivery, which is conducive to reducing the toxic side effects of drugs.

### Bacterial outer membrane vesicles

Bacterial membrane vesicles are membranous bodies secreted by bacteria and contain thalli active substances. As a class of bacterial membrane vesicles, bacterial OMVs have been widely explored, and they are spherical bilayer nanolipids that are released by gram‐negative bacteria, ranging from 20 to 300 nm.^67^, ^68^

Firstly, the characteristics of the parental bacteria are inherited, so vesicles can target tumor cells.^69^ Additionally, vesicles, containing lipopolysaccharides, pathogen‐related proteins and lipoproteins, are highly immunogenic, so they can stimulate the natural immune system.^70^ At present, vesicles are expected to act as carriers and immune adjuvants and be widely used in vaccine platforms.^71^ Secondly, vesicles can enter the host cell by directly fusing with the host cell membrane or by endocytosis. For instance, OMV can be taken up by infected cells and effectively inhibit the intracellular survival of *Staphylococcus aureus*.^72^ Finally, they are less virulent than bacteria and can be absorbed by cells.

Outer membrane vesicles provide an alternative solution to the dilemma of poor drug delivery efficiency for refractory diseases. In the drug delivery system for non‐small cell lung cancer, OMV delivers the strongest intracellular DOX fluorescence intensity, which indicates that OMV significantly improves the efficiency of DOX delivery to tumor cells.^73^ OMV has promoted the rapid development of tumor vaccines. Combining gene engineering and molecular glue technology, a personalised tumor mRNA vaccine using OMVs was constructed.^74^ The OMV‐based vaccine induces a complete regression of 37.5% in the CRC model and produces a stronger immune response when compared to the liposome‐based vaccine. Besides, OMV facilitates the development of cancer vaccines with short treatment cycles, which is expected to reduce the financial burden on patients.^75^ Basic fibroblast growth factor (BFGF), a tumor‐promoting factor, plays an important role in angiogenesis, proliferation and invasion of tumor cells, resistance to apoptosis, and tumor immunity.^76^, ^77^, ^78^ In mouse models of lung tumors, BFGF‐loaded OMV treatment resulted in complete tumor suppression in 50% of the mice, with elevated levels of BFGF antibodies persisting for a period following three immunisations.

## Applications of synthetic biology, chemistry and nanotechnology to functionalised and engineered bacteria

In bacteria‐based drug delivery systems, synthetic biology technologies provide an alternative way to customise bacteria with tumor‐targeting capabilities for efficient drug delivery. Besides, the applications of chemical and nanotechnology, including surface modification and electrostatic adsorption, have facilitated bacterial drug loading and protected the drug‐carrying bacteria before reaching the target site to avoid premature drug leakage and enhance delivery efficiency.

### Synthetic biology

Genome editing and gene circuits that are representative of synthetic biology have been applied to bacteria‐based drug delivery systems. Traditional tumor therapies suffer from the lack of tumor targeting, low concentration of drugs at the tumor site, and resistance of tumor stem cells to various therapies. Engineered bacteria modified by synthetic biology are expected to overcome these problems.^79^

#### Genome editing

**Figure 2:**
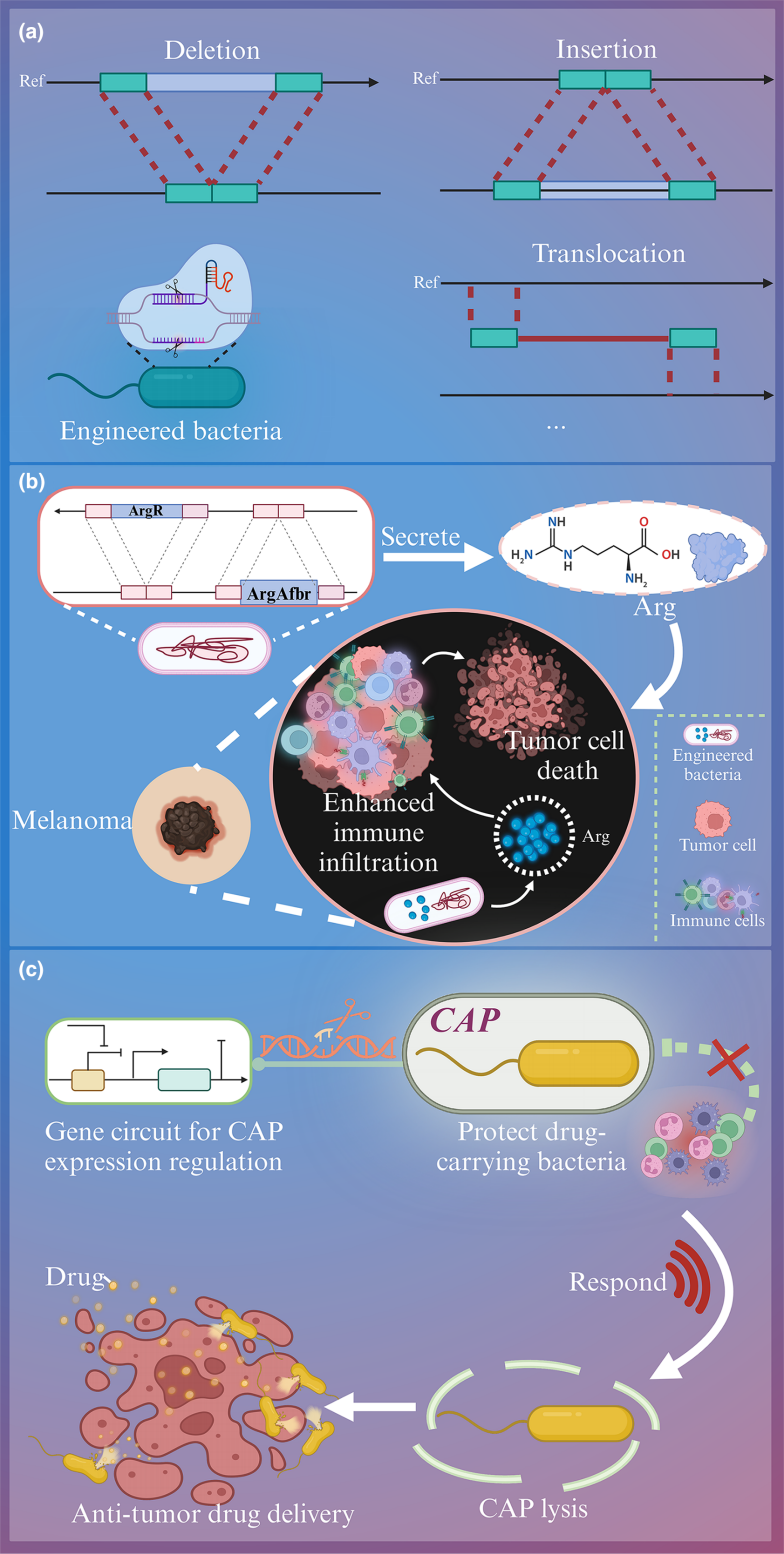
Applications of synthetic biology to functionalized and engineered bacteria. **(a)** Common genome editing methods for engineered bacteria. **(b)** Construction of engineered bacterial L‐Arg with the ability to synthesise anticancer substances. **(c)** Programmable bacterial surface capsular polysaccharide (CAP) expression system.

Bacteria are genetically engineered to efficiently express exogenous genes by specifically adding, deleting and altering genetic material to become engineered bacteria (Figure 2a). Engineered bacteria can inhibit tumor growth, regulate gut bacteria, and alleviate chemically induced dysbiosis.^80^ For instance, intracellular L‐arginine concentration directly affects the metabolic adaptability and viability of T cells, and it is essential for anti‐tumor response.^81^ L‐arginine (L‐Arg) bacteria that direct ammonia for arginine synthesis were synthesised^82^ (Figure 2b). The arginine repressor gene (ArgR) of *E. coli* was deleted and a resistant mutant ArgAfbr that was not repressed by L‐Arg was integrated into the bacterial genome. L‐Arg bacteria promoted the accumulation of arginine at a high concentration and had the best anti‐tumor effect when combined with PD‐L1 antibody. The engineered bacteria enhance the anti‐tumor immune response by regulating the tumor microenvironment. Additionally, gene engineering can also create a built‐in biological ‘kill switch’ to prevent the bacteria from surviving *in vitro*.^83^

Targeted gene insertion or replacement is an effective gene editing tool for gene engineering. Currently, many researchers have revealed that combining anti‐tumor drugs or acoustic sensitisers with focused ultrasound therapy can significantly improve the therapeutic effect.^84^ An ultrasound‐responsive bacterium (URB) has been constructed by gene replacement and insertion plasmid recombination. In a 4T1 tumor mouse model, URB combined with ultrasound treatment inhibits tumor metastasis to distant areas and extends survival time (median survival time 60 days), which provides a new idea for bacteria‐mediated tumor therapy.^85^ Moreover, the engineered bacteria that expressed tumor growth inhibiting nano antibody and temperature‐dependent genes was developed.^86^ Combined with focused ultrasound technology, engineered bacteria can be applied for targeted treatment of deep‐seated tumors.

#### Gene circuits

Recently, engineered bacteria have been used for anti‐tumor drug delivery through synthetic genetic circuits. In bacteria‐based drug delivery systems, the gene circuit can be divided into three basic parts, namely input, operation and output.^87^ First, the drug‐carrying bacteria can sense and interpret the biological or abiotic signals of the environment or disease, such as pH, temperature and metabolites through sensing elements. Second, the expected gene expression results were generated by computing and processing appropriate bacterial behaviour based on molecular signals through genetic logic gates. Finally, the engineered bacteria were driven to perform the expected therapeutic behaviour, such as time, to release the drug.^88^

The gene circuit controls the release of drugs delivered by bacteria, which is of great significance for the efficiency and toxicity of drug delivery. A *Salmonella*‐based intracellular delivery system was designed to deliver the central domain of nuclear inhibitor of protein phosphatase 1 (NIPP1‐CD) and constitutive two‐chain active caspase‐3 (CT Casp‐3) that are protein drugs that induce apoptosis for the treatment of solid tumors.^12^, ^89^, ^90^, ^91^ The system‐derived *Salmonella* invasion of tumor cells through the regulator flhDC and released the protein drug by triggering the promoter *Salmonella* pathogenicity island 2 to cleave the bacteria. The administration of NIPP1‐CD and CT Casp‐3 via *Salmonella* substantially suppresses tumor cell growth and metastasis in *in vitro* cultured cell model and 4T1 tumor mouse model. The *Salmonella*‐based delivery platform has increased the delivery of protein drugs by more than 500‐fold, with great potential for application in poorly treated tumors.

In recent years, gene circuits have acquired great potential to prevent premature degradation of drug‐carrying bacteria. *EcN* is often used in tumor treatment as a probiotic, with clinical therapeutic effects in live bacterial therapeutic.^92^ The programmable bacterial surface capsular polysaccharide (CAP) expression system that sensed and responded to induce stimuli and regulate cell surface properties was constructed^93^ (Figure 2c). CAP can accomplish bacterial immune escape, and the removal of CAP allows for the clearance of bacteria. CAP increased the maximum tolerated dose of *EcN* 10‐fold and significantly inhibited tumor growth in CT26 CRC and MMTV‐PyMT mouse models. This system provides a new direction to achieve microbial immune escape by controlling the expression of surface capsular polysaccharides outside the bacteria through gene circuits.

The Quorum sensing bacteria obtained by gene engineering release anti‐tumor drugs through genetic circuitry lysis, which provides systemic exposure to drugs and exerts powerful anti‐tumor effects.^94^, ^95^ The *Salmonella*‐based synchronous lytic circuit (SLC) achieves a high level of apoptosis and death of tumor cells in subcutaneous tumor models and extends survival time of mice with metastatic CRC.^23^ The modified *Salmonella* produces and accumulates N‐lipoylhomoserine lactone to a threshold value through a positive feedback loop. Upon reaching a threshold, SLC can be triggered, which could activate the quorum‐sensing bacteria pathway and trigger bacterial lysis or rupture that releases the therapeutant.

### Chemistry and nanotechnology

**Figure 3:**
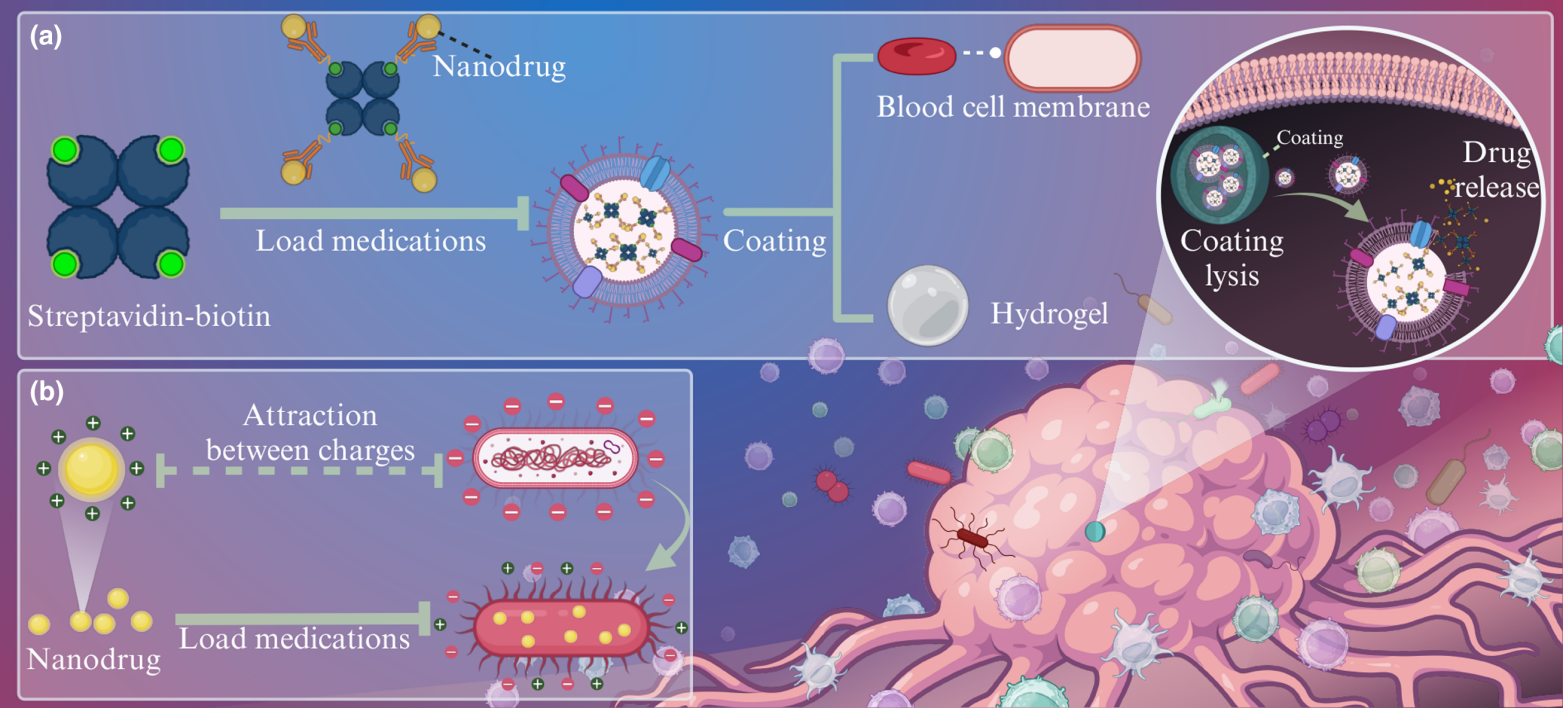
Applications of chemistry and nanotechnology in bacteria‐based drug delivery systems. **(a)** Application of surface modification for drug loading and protection of drug‐carrying bacteria. **(b)** Application of electrostatic adsorption for drug loading.

The rapid development of chemical and nanotechnology has provided great support for the customisation of efficient drug delivery bacteria. Surface modification changes the properties or functions of the surface of a material by chemical treatment, physical treatment, or coating. Here, surface modification is applied to adsorb anti‐tumor drugs to the bacteria and provide a coating to protect the drug‐carrying bacteria (Figure 3a). In addition to surface modification and synthetic biology techniques, positively charged nanomaterials can be used to load drugs to bacteria through electrostatic adsorption in a convenient manner (Figure 3b).

#### Surface modification

Bacterial cell wall composition is complex and chemical groups, such as carboxyl and amine, offer the possibility to connect bacteria with multifunctional nanoparticles. As a non‐covalent strong effector, streptavidin‐biotin is widely used in drug delivery systems to connect bacteria with functional nanoparticles, such as photosensitisers and magnetic particles.^96^, ^97^

Bacterial surfaces are coated or modified by surface modifications to protect bacteria from drug leakage and enhance the ability of bacteria to target tumors. The use of synthetic materials, such as liposomes and hydrogels, to encapsulate bacteria can prevent the premature removal of drug‐carrying bacteria to some extent. For example, the survival rate of *EcN* with complete fibroin protein coating in simulated gastric juices was increased by nearly 52 times after four cycles of deposition.^98^ A significant reduction in the expression level of the tumor necrosis factor TNF‐α with silk‐coated *EcN* treatment was obtained in a mouse model of intestinal mucositis, with the optimal therapeutic effect. The silk coating is expected to protect the drug‐loaded bacteria in the gastrointestinal tract from premature bacterial degradation and drug leakage.

Furthermore, nanopolymer can be removed in response to *in vivo* biological signals with the help of the gene circuit, thereby allowing the bacteria to function at specific sites.^99^, ^100^ However, it still activates the body's complement system, thus leading to allergic reactions. The blood cell membranes can be used to wrap the drug‐carrying bacteria.^101^ In this way, the clearance capacity of the body's immune system can be significantly reduced to increase the enrichment of bacteria at the tumor site.

#### Electrostatic adsorption

The mechanism of electrostatic adsorption is that ions or groups with different charges attract each other under the guidance of electrostatic gravity, so that dissimilar charges or groups are combined.^102^ Electrostatic adsorption is widely used in drug delivery systems to connect drugs and carriers for its simplicity of operation. Besides, electrostatic adsorption does not involve the use of organic solvents, thereby eliminating the generation of any residual organic solvents during the drug inclusion process. Therefore, the drug‐carrying preparations prepared by this method are relatively stable. Gene drugs, which are easily destroyed by enzymes and other substances in the body without a drug delivery system, are often connected to gene carriers by electrostatic adsorption.

As mentioned earlier, the surface of bacteria is usually negatively charged, which provides an opportunity to construct drug‐loaded bacteria by electrostatic adsorption. Wei et al. constructed a polarised nanoparticle‐loaded bacterial system based on *E. coli*. *E. coli* and PR848 (drug‐carrying nanoparticles) were connected with ethylene glycol chitosan to prepare PR848 nanoparticle‐load *E. coli* (Ec‐PR848) by electrostatic interaction.^103^ It was discovered that the maximum area of the cell nuclear proliferation and cell necrosis, maximum area of apoptosis, and minimum area of proliferation were in Ec‐PR848‐treated mice cells. The drug delivery system constructed via electrostatic adsorption has the advantage of simple preparation, which could strongly support the promotion of highly effective anti‐tumor system.

## Prospects and challenges

Conventional drug delivery methods, such as tablets and capsules, are widely used in clinical practice because of their simple preparation and high compliance, but there are many shortcomings in the delivery of anti‐tumor drugs.^104^, ^105^ While common small molecule anti‐tumor drugs exert anti‐tumor effects, they also have strong toxicity to normal tissues. However, traditional drug delivery systems lack specific targeting, thus resulting in non‐specific drug distribution.^106^ After blood circulation, anti‐tumor drugs are dispersed in different tissues. In this way, not only the drug concentration at the tumor site is reduced, but also the toxic side effects are increased. In addition, the physicochemical properties of antineoplastic drugs, such as solubility and stability, also put forward higher requirements for drug delivery methods. For example, camptothecin antineoplastic drugs, such as topotecan and irinotecan, have strong anticancer effects.^107^ Unfortunately, these drugs are poorly water‐soluble and easily decomposed in light, and the lactone ring, an essential group for anti‐tumor activity, is easily inactivated under physiological conditions. Therefore, conventional delivery methods lead to low drug delivery efficiency and marked toxic side effects.

The core of bacteria‐based drug delivery systems is that bacteria, as living organisms, are used as carriers of anti‐tumor drugs because of their potential to target and enrich at the tumor site, providing an important prerequisite for the application of synthetic biology, chemistry and nanotechnology. Bacteria can be used as carriers in various forms, such as BGs, OMVs and minicells, which provides a broad platform for the application of technology. Unlike bacteria, other nano‐drug carriers, such as liposomes, must be tethered to technology to achieve tumor targeting.^104^ In bacteria‐based drug delivery systems, although the application of various technologies plays an important role in optimising the bacteria‐based drug delivery systems, leading to the enhanced the anti‐tumor effect, bacteria are more critical as active anti‐tumor drug carriers. With the support of synthetic biology, chemistry and nanotechnology, bacteria‐based drug delivery systems have certain advantages over traditional drug delivery methods, because they can ultimately increase the accumulation of drugs at the tumor site and reduce the toxic side effects of drugs. First, bacteria can achieve targeted drug delivery to the tumor site, thus avoiding the non‐specific distribution of drugs.^108^ Furthermore, bacteria are easy to culture and store, and the transformation into engineered bacteria is simple and feasible through genetic engineering. Scalable bacterial culture technology is expected to enable more cost‐effective mass production compared with other novel drug carriers. Additionally, synthetic biology techniques, such as gene circuits, allow the controlled release of drugs at tumor sites through the genetic modification of bacteria.^109^, ^110^ Fu and colleague*s* constructed a genetic circuit to program the bacterial lifestyle in response to NIR light to precisely control drug release based on the attenuated *Pseudomonas aeruginosa* (*P. aeruginosa*) via gene knockout.^111^ After three cycles of tumor injection‐illumination treatment, drug release from the engineered bacteria induced 30% of tumor regression and significantly inhibited the remaining tumor growth in the A549 tumor‐bearing mouse model.^111^ In this way, the early release of the drug is not only avoided but also expected to achieve a sustained slow release of the anti‐tumor drug, eventually leading to a significant enhancement of the anti‐tumor effect. Finally, in addition to their ability to efficiently deliver anti‐tumor drugs, some bacteria, such as magnetotactic bacteria AMB‐1, can also inhibit tumor growth by themselves,^112^ thus achieving enhanced anti‐tumor effect in combination with drugs.

Nevertheless, the future of bacteria‐based drug delivery systems for clinical translation is challenging because of several limitations. First and foremost, potential biosafety hazards in bacteria‐based drug delivery systems are the greatest concern and have been a major impediment to regulatory approval. Except for a few bacteria (generally recognised as safe organisms ‘GRAS’) that can be directly used in tumor treatment among natural bacteria, most bacteria can cause bacterial infections and bring biosafety concerns. The accumulation of bacteria can cause adverse reactions, such as allergic and inflammatory responds.^113^ Most patients with malignant tumors have low immunity, and even the residual virulence of bacteria after drug delivery may do harm to patients. Therefore, more studies on the bacterial biosafety need to be conducted to find the optimal bacterial loading concentration with high drug delivery efficiency and low bacterial toxicity. Attenuated engineered strains have been reported in previous studies and perform well *in vivo*.^12^ However, there are still differences in pharmacokinetics and pharmacodynamics between human and mouse tumors. Therefore, the future evaluation of bacterial virulence is more suitable in the xenograft mouse model. Additionally, human microecological disorders may be induced by the involvement of drug‐carrying bacteria. In view of the close relationship between microecological dysbiosis and the occurrence of diseases,^114^ it is necessary to consider the effect of drug‐carrying bacteria on human microecology in subsequent studies. Finally, genetic contamination should be given more attention because of the possibility of engineered bacteria invading the human genome, which raises serious ethical concerns. CRISPER/CAS9 that is commonly used in engineered bacteria has been reported to have the possibility of driving gene contamination.^115^ Once genetic contamination occurs, it can proliferate and spread rapidly.

## Conclusion

Bacteria are closely associated with cancer progression. Not only does bacteria target tumors, but it has a strong potential for gene engineering as an active organism. The traditional drug delivery method currently used in clinical practice leads to low efficiency of anti‐tumor drug delivery and high toxicity. Recently, bacteria or bacterial secretions as carriers for drug delivery made full use of the tumor‐targeting ability of bacteria. Synthetic biology, chemistry and nanotechnology techniques used in drug delivery systems provide a platform for loading drugs onto bacteria and preventing their premature degradation. Currently, most of the research remains in the preclinical stage, and it is challenging to achieve clinical translation. The potential biosafety hazards, regulatory hurdles and ethical considerations need to be addressed in bacteria‐based drug delivery systems. In conclusion, the involvement of bacteria into drug delivery systems holds the potential to enhance drug delivery efficiency and augment the therapeutic impacts of anti‐tumor drugs and deserves further investigation in the future.

## Author contributions

**Han Shuwen:** Writing – original draft. **Song Yifei:** Writing – original draft. **Wu Xinyue:** Data curation. **Qu Zhanbo:** Visualization. **Yu Xiang:** Conceptualization. **Yang Xi:** Conceptualization.

## Conflict of interest

The authors declare no conflict of interest.
